# Esophagopericardial Fistula After Esophagectomy

**DOI:** 10.7759/cureus.13753

**Published:** 2021-03-07

**Authors:** Yasmin Khader, Sami Ghazaleh, Christian Nehme, Jordan Burlen, Ali Nawras

**Affiliations:** 1 Department of Internal Medicine, University of Toledo, Toledo, USA; 2 Division of Gastroenterology and Hepatology, University of Toledo, Toledo, USA

**Keywords:** esophageal cancer, esophagectomy, esophagopericardial fistula, cardiac tamponade, upper endoscopy

## Abstract

Esophagectomy is the mainstay surgical treatment for esophageal carcinoma. The operation can be complicated by an anastomotic stricture, anastomotic leak, recurrent laryngeal nerve injury, conduit ischemia, and chylothorax. Rarely, esophagectomy can be complicated by fistula formation between the gastrointestinal tract and the nearby structures. We describe a case of esophagopericardial fistula after esophagectomy. A 50-year-old man presented to the emergency room with a chest pain of two-week duration associated with sweating, chills, and poor appetite. He was diagnosed with stage III esophageal adenocarcinoma four months ago. He had received neoadjuvant chemotherapy followed by distal esophagectomy, partial gastrectomy, and placement of a jejunostomy tube one month before presentation. Cardiovascular examination was significant for jugular venous distention and distant heart sounds. Chest CT angiography showed pneumomediastinum concerning for anastomotic leak. Esophagram finally confirmed an esophagopericardial fistula. A drain was placed into the pericardial space followed by emergent esophageal stent placement. Eventually, he underwent a cervical esophagostomy and placement of a jejunostomy tube. The patient was later discharged home in a stable condition. In conclusion, esophagopericardial fistula is a rare adverse event of esophagectomy. Esophageal stenting could be useful as a temporary or definite treatment.

## Introduction

Esophagectomy is the mainstay surgical procedure used in the management of esophageal cancer [1]. This operation is technically challenging and may result in a wide range of complications. Pulmonary and cardiac complications are the most common and include pneumonia, pulmonary embolism, acute respiratory distress syndrome, atrial fibrillation, and myocardial infarction [2,3]. Procedure-specific complications include anastomotic stricture, anastomotic leak, recurrent laryngeal nerve injury, conduit ischemia, and chylothorax [4,5]. Rarely, esophagectomy can be complicated by fistula formation, which connects the gastrointestinal (GI) tract with the surrounding structures. Case reports in the literature describe fistulas that connect the esophagus or stomach to the airway, aorta, pleura, or pericardium [6-8]. In this report, we describe a case of esophagopericardial fistula after esophagectomy.

This case was presented as an abstract at the 2020 American College of Gastroenterology meeting.

## Case presentation

A 50-year-old man presented to the emergency room with chest pain of two-week duration. The chest pain was diffuse and radiated to both shoulders. It was dull in character and progressively worsened. He rated the severity of the pain as 6 out of 10 per the visual analog scale. He could not identify any exacerbating or relieving factors. His chest pain was associated with sweating, chills, and poor appetite. He denied shortness of breath, cough, dysphagia, abdominal pain, nausea, vomiting, diarrhea, or constipation.

The patient’s past medical history was significant for moderately differentiated stage III (T3N1M0) esophageal adenocarcinoma diagnosed four months ago. He had received neoadjuvant chemotherapy (cisplatin and fluorouracil) and radiation therapy followed by distal esophagectomy, partial gastrectomy, and placement of a jejunostomy tube one month before his current presentation. The surgery was complicated by right-sided pneumothorax, which resolved after chest tube placement. At that time, an esophagram was performed, which showed no evidence of an anastomotic leak. His other medical problems included anxiety and anemia of chronic disease. Family history was positive for bronchogenic carcinoma in his mother and brother. He was an active smoker who smoked 10.5 pack-years. He denied alcohol and illicit drug use.

On physical examination, the patient appeared cachectic and diaphoretic. Vital signs demonstrated a blood pressure of 93/73 mmHg and a heart rate of 175 beats per minute. The remaining vital signs were normal with a body temperature of 36.6°C and a respiratory rate of 18 breaths per minute. Cardiovascular examination was significant for tachycardia, jugular venous distention, and distant heart sounds. Lung examination demonstrated bilateral clear lungs and no crackles or wheezes. The abdomen was soft and non-tender with normal bowel sounds. Complete blood count revealed low hemoglobin of 8.6 g/dL. He also had elevated white blood cells of 21.1 × 10^9^/L and platelets of 871 × 10^9^/L. Comprehensive metabolic panel showed low sodium of 130 mmol/L and albumin of 2.8 g/dL.

**Figure 1:**
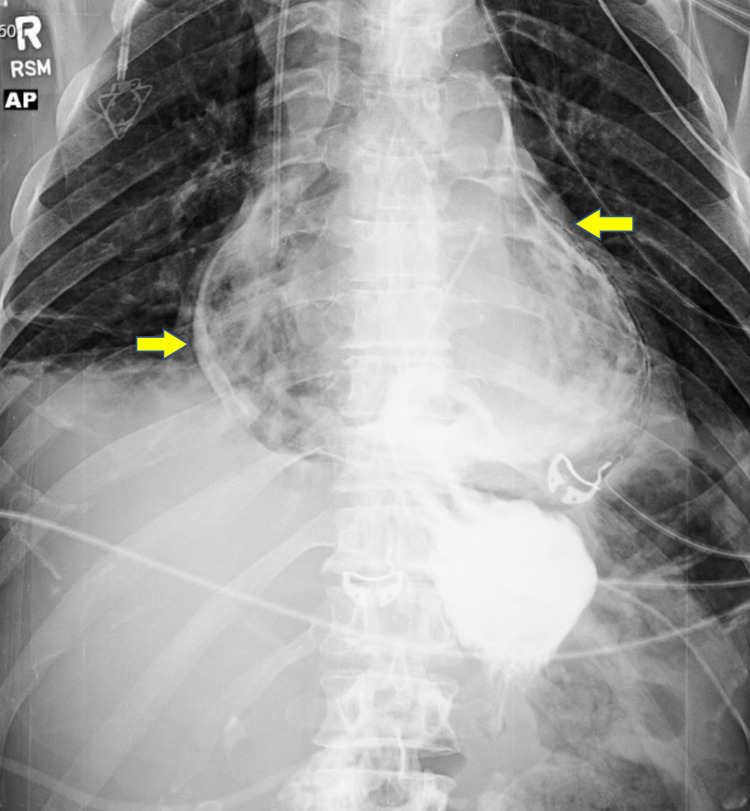
Esophagram showing contrast leaking from the esophagus into the pericardium (arrows), consistent with an esophagopericardial fistula

Initial chest X-ray showed bilateral pleural effusions with parenchymal opacities in both lower lung fields, suggestive of atelectasis. It did not show cardiomegaly or widening of the mediastinum. The EKG was only significant for sinus tachycardia, and troponin was within normal limits. Chest computed tomography angiography (CTA) was significant for pneumomediastinum, which was concerning for anastomotic leak. An esophagram was then performed, which confirmed the presence of contrast leak at the gastroesophageal junction communicating directly with the pericardium (Figure 1).

**Figure 2:**
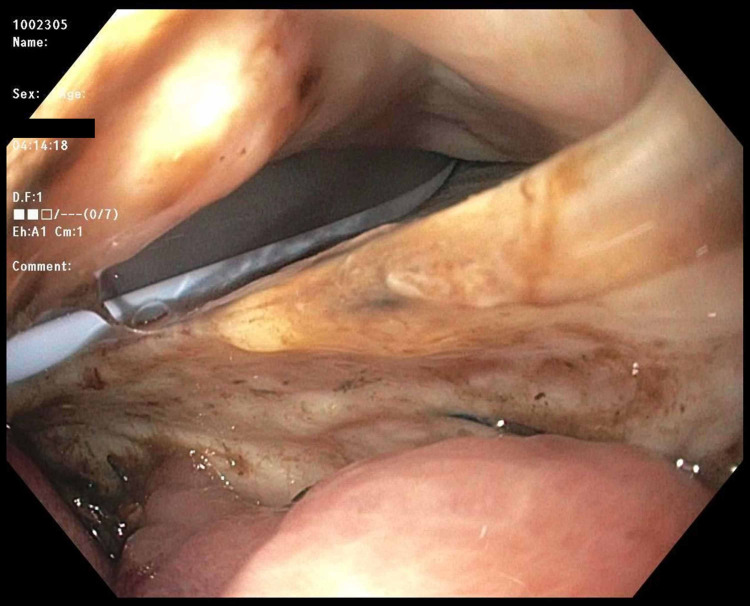
Esophagogastroduodenoscopy showing esophagopericardial fistula with pericardial draining catheter seen within the esophageal lumen

**Figure 3:**
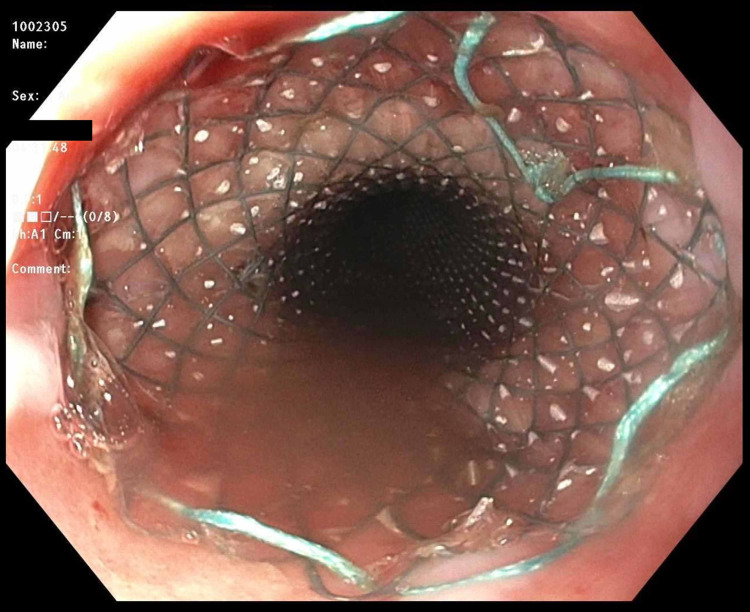
Esophagogastroduodenoscopy showing the esophageal stent in place across the fistula site

**Figure 4:**
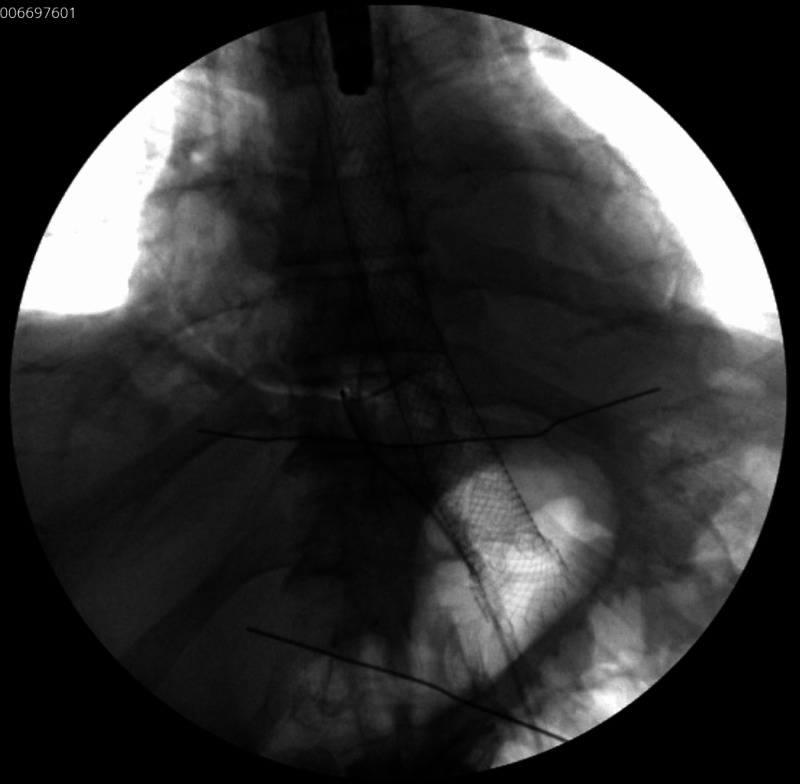
Fluoroscopic confirmation of esophageal stent placement

The patient was stabilized with intravenous fluids and antimicrobials including ceftriaxone, metronidazole, and fluconazole. Immediately after, a pericardial window was performed for diagnostic and therapeutic purposes. The procedure revealed thickening and inflammation of the pericardium. It was followed by drain placement, which evacuated nonclotting blood combined with obvious enteric contents including partially digested food. Simultaneously, an esophagogastroduodenoscopy (EGD) was performed, which confirmed the presence of a 25-mm esophagopericardial fistula at the gastroesophageal anastomosis site. The pericardial draining catheter was seen within the esophageal lumen (Figure 2). The endoscopist then placed a fully covered self-expandable metallic esophageal stent across the gastroesophageal anastomosis site (Figure 3). No strategies were used to prevent migration of the stent. The position of the stent was confirmed endoscopically and fluoroscopically (Figure 4). The patient tolerated the procedure well and was sent to the intensive care unit in a stable condition. The patient continued to have biliary drainage from the pericardial drain indicating a persistent leak. An esophagram was performed, which confirmed the persistence of anastomotic leak with inability of the stent to seal distally. The patient and his family had a long discussion with the surgeon about the treatment options. The patient eventually underwent a complete resection of the remaining portion of the esophagus with cervical esophagostomy and placement of a jejunostomy tube. The postoperative course was unremarkable, and he was discharged home in a stable condition. Unfortunately, the patient passed away due to septic shock secondary to community-acquired pneumonia.

## Discussion

Esophagopericardial fistula is an acquired abnormal connection between the esophagus and the pericardium [8]. It is an extremely rare complication, with an overall mortality rate of more than 50% to 80% [9]. Benign esophageal disorders are the most common underlying etiology of this complication. Around 35% of all cases are due to chronic esophagitis, often associated with acid reflux and strictures. Foreign body ingestion with secondary perforation is the second most common cause, accounting for 16% of cases [7]. Other recognized causes are esophageal carcinoma (24% of all cases), penetrating or blunt thoracic trauma, and iatrogenic causes such as catheter ablation for atrial fibrillation, upper endoscopy, and esophagectomy (6% of all cases) [10]. The most feared outcomes of these fistulas are purulent pericarditis and cardiac tamponade [7]. Early diagnosis and treatment with pericardial drainage and appropriate surgical intervention are essential for survival.

Clinically, patients with esophagopericardial fistula can be asymptomatic or present with various complaints. The symptoms range from chest pain and shortness of breath to sudden unexpected death secondary to cardiac tamponade [7]. Key elements, which determine progression to cardiac tamponade and cardiovascular collapse, are the rate of air accumulation relative to pericardial compliance and the presence of intact compensatory mechanisms. In our case, the patient complained of chest pain referred to both shoulders, suggestive of pericardial inflammation, with possible diaphragmatic irritation being the cause of the symptoms. He also had hypotension, elevated jugular venous pressure, and distant heart sounds on physical examination concerning for cardiac tamponade. Other possible clinical findings are pulsus paradoxus and subcutaneous emphysema.

Diagnosis can be reached using simple erect plain radiography that commonly shows pneumopericardium in 50% of cases [11]. In our case, pneumopericardium was detected by chest CTA. Once a fistula is suspected based on the initial findings, an esophagram is the appropriate next step to confirm the diagnosis. Esophagram can detect 80% of these fistulas through demonstrating gross filling of the pericardial sac with contrast material used in upper GI contrast studies. Upper endoscopy is also useful but it should be used with caution, as excessive air insufflation during the procedure may exacerbate the cardiac tamponade [12]. In our case, a large pericardial fistula of 25 mm was demonstrated at the gastroesophageal anastomosis site with a pericardial draining catheter seen within the esophageal lumen.

The management of esophagopericardial fistula is significantly improving especially in cases with early detection and timely appropriate intervention. Many techniques have been used in the management of esophagopericardial fistula, including simple drainage with the acceptance of a prolonged fistula. This conservative management would include a pericardial window, multiple drainage tubes, and systemic antibiotics. This remains a valuable option for patients with disseminated malignancy or those who are poor surgical candidates [13]. Another option is the placement of a soft large-bore T-tube through the perforation to create a controlled fistula [14]. Alternatively, these fistulas can be managed by immediate esophagogastric resection with re-anastomosis, particularly if there is a distal obstructive lesion and/or neoplasm [15]. These methods and their modifications have all been employed with varying success.

In our case, a temporary esophageal stent was placed to stabilize the patient and minimize further drainage into the pericardium. This technique is commonly used for esophagopericardial fistulas complicating catheter ablation procedures for atrial fibrillation [16]. Usually, an esophageal stent is used as a temporary measure before definite surgical intervention. However, Eitel et al. described a case series where three patients with catheter ablation-induced esophagopericardial fistulas were treated with esophageal stenting without surgical intervention [17]. The esophageal stents were removed in one to two months, and the patients never required surgical treatment.

Esophagopericardial fistulas can occur as complications of esophageal cancer. Tombazzi et al. described the case of a patient who underwent successful esophageal stenting without needing surgical treatment [18]. The patient was discharged home in a stable condition but died five weeks later while in hospice for reasons unrelated to the esophageal stent. Dy et al. described another case of esophagopericardial fistula complicating esophageal cancer and varices [19]. The patient was treated with esophageal stent placement that was later replaced with another stent due to a persistent leak. However, the patient decided against further treatment and died while in hospice of a respiratory illness.

To our knowledge, Shahian and Kittle reported the only case with esophagopericardial fistula occurring as a complication of esophagectomy for esophageal cancer [14]. The patient was treated successfully with T-tube placement into the fistula, which was removed four weeks later. Dafnios et al. described a similar case of esophagopericardial fistula complicating gastrectomy for esophageal cancer [20]. Although the fistula was not confirmed with an esophagram, an echocardiogram revealed a small hydropneumopericardium. The patient was treated with a conservative approach given the small size of the hydropneumopericardium. In addition, treatment included antibiotics and chest tube placement to treat an associated pleural effusion. Unfortunately, the patient deteriorated and died five weeks later.

The use of esophageal stenting appears to be successful in treating esophagopericardial fistulas complicating atrial fibrillation catheter ablation and esophageal cancer. However, we could not find a case where esophageal stenting was used for an esophagopericardial fistula complicating esophagectomy. The two cases reported by Shahian and Kittle and Dafnios et al. described fistulas that occurred after esophagectomy and gastrectomy, respectively. Both patients were managed conservatively and did not undergo stent placement or surgical treatment. In our case, we describe esophageal stent placement for an esophagopericardial fistula that complicated esophagectomy. An esophageal stent was placed as a temporary therapy before the patient underwent surgical resection. This proved to be a good strategy as it stabilized the patient’s condition until the definitive management. Alternatively, we could have replaced the esophageal stent with another stent, as in the case of Dy et al. It is unclear whether this technique would have resulted in a better seal and obviated the need for surgery.

## Conclusions

Esophagopericardial fistula is a rare occurrence that can complicate esophagectomy. It can result in cardiac tamponade and therefore can be highly lethal. A good history and physical examination can identify this life-threatening condition. Once the diagnosis is reached, the placement of an esophageal stent could be an option to stabilize the patient. Although esophageal stenting failed in our patient, further studies could examine the role of esophageal stents as a temporary therapy before surgical intervention. Moreover, we should examine their role as possible definitive treatment for esophagectomy-induced fistulas given their success in treating fistulas complicating atrial fibrillation catheter ablation and esophageal cancer.
